# 47 Successful Prevention of Secondary Burn Progressions Using Topical Tacrolimus and Infliximab Hydrogel

**DOI:** 10.1093/jbcr/irac012.050

**Published:** 2022-03-23

**Authors:** Christopher L Kalmar, Colin G White-Dzuro, Alonda Pollins, Brady Burns, Patrick Assi, Harrison Thomas, Kianna Jackson, Galen Perdikis, Lleon Bellan, Wesley Thayer

**Affiliations:** Vanderbilt University Medical Center, Nashville, Tennessee; Department of Biomedical Engineering, Vanderbilt University, Nashville, TN, USA, Nashville, Tennessee; Vanderbilt University Medical Center, Nashville, Tennessee; Meharry Medical College, Nashville, Tennessee; Vanderbilt University Medical Center, Nashville, Tennessee; Vanderbilt University Medical Center, Nashville, Tennessee; Vanderbilt University Medical Center, Nashville, Tennessee; Vanderbilt University Medical Center, Nashville, Tennessee; Vanderbilt University Medical Center, Nashville, Tennessee; Vanderbilt University Medical Center, Nashville, Tennessee

## Abstract

**Introduction:**

The pathophysiology of partial- to full-thickness burn wound conversion remains poorly understood. Recent studies have demonstrated that an altered inflammatory response may play be implicated in this secondary conversion to deeper wounds. Therefore, reduction in early inflammation may decrease burn severity and morbidity.

Specifically, TNF-ɑ has been shown to detrimentally affect the healing process after injury through a variety of mechanisms. We hypothesized that microcapillary alginate hydrogel loaded with immunosuppressive medications applied to partial-thickness burns would reduce inflammation and prevent further progression to full-thickness burns.

The purpose of this study was to determine whether topical application of infliximab or tacrolimus could decrease burn wound depth.

**Methods:**

Assembly of the microfluidic hydrogels was achieved by embedding microfibers within a hydrogel scaffold composed of an alginate blend. The treatment cohorts received either (1) infliximab loaded hydrogel or (2) tacrolimus skin ointment covered by hydrogel. The control cohort only received an occlusive dressing.

There were 12 young (2-4 months) and 12 old ( >16 months) mice, which were separated into treatment and control cohorts. All mice were anesthetized and given partial thickness burns by a validated scalding protocol. Mice were euthanized on post-burn day 3, and skin samples were taken. Burn depth was evaluated using Vimentin immunostaining.

**Results:**

In young mice, infliximab hydrogel (p=.002) and tacrolimus hydrogel (p=.002) significantly decreased burn depth compared to controls. In old mice, infliximab hydrogel (p=.005) and tacrolimus hydrogel (p< .001) significantly decreased burn depth compared to controls.

In young mice, infliximab and tacrolimus were similarly efficacious (p > .05). In old mice, tacrolimus significantly decreased burn depth compared to infliximab (p=.002).

In controls, old mice had deeper burn wound progression than young mice (p< .001). Similarly, in those treated with infliximab, old mice had deeper burn wound progression than young mice (p=.002). Interestingly, tacrolimus was able to decrease burn wound depth in old mice such that their burn wound thickness was similar to young mice (p >.05).

**Conclusions:**

Application of a novel microcapillary alginate hydrogel infused with infliximab or topical tacrolimus reduced partial- to full-thickness burn wound conversion in mice. Application of immunosuppressive dressings may be a promising avenue for further clinical investigation to reduce morbidity and mortality associated with burn injuries.

